# Suicidal tendencies and its association with psychoactive use predictors among university students in Uganda: a cross-sectional study

**DOI:** 10.4314/ahs.v21i3.53

**Published:** 2021-09

**Authors:** Sheila Wesonga, Charles Osingada, Allen Nabisere, Stanley Nkemijika, Connie Olwit

**Affiliations:** 1 Makerere University College of Health Sciences, Department of Nursing; 2 Georgia State University, Public Health

**Keywords:** Suicidal tendencies, psychoactive use, university students, Uganda

## Abstract

**Background:**

Globally, suicide is one of the leading causes of death, and approximately 80% of all suicides occur in lowand middle-income countries. Younger people in Africa are at a higher risk of suicide than others.

**Objective:**

To describe the prevalence and factors associated with suicidal tendencies among undergraduate university students using alcohol and other psychoactive substances.

**Methods:**

Convenient sampling was used to identify 400 students who participated in the study. Socio-demographic and Mini-International Neuropsychiatric Interview tools were used to obtain information. Data were analyzed using SAS 9.4 and presented in descriptive and inferential statistics.

**Results:**

Among the respondents, 80% were male, and 85% were using marijuana. 6.3% had suicidal tendencies. Respondents from the northern region had more suicidal tendencies than other regions, and unemployed students had more suicidal tendencies than those employed. After multivariate analysis, being abusive and dependent on other psychoactive substances was associated with suicidality. And having dependence on both alcohol and other psychoactive substances was associated with suicidality.

**Conclusion:**

Suicidality screening and psychosocial support should be provided to this vulnerable population. Efforts There is a need to strengthen, implement more effective preventive strategies to reduce the use of alcohol and other psychoactive substances.

## Introduction

Suicidality is a global public health issue. Suicidality is defined as wishing to be dead, suicidal ideation, suicide planning, and attempted suicide1. Globally, suicide is one of the leading causes of death. In 2016, WHO estimated 800,000 deaths were by suicide worldwide^2^.There are indications that for every one adult who dies of suicide, there could have been 20 others who attempt suicide^3^. Approximately 80% of all suicides occur in lowand middle-income countries^4^. Furthermore, in 2016 in Uganda, about 9.9 per 100,000 population died because of suicide^2^.

Despite high global mortality rates, suicide rates in Africa have been meager^5^. In African societies, suicide is a taboo, and a failed suicide attempt is punishable by law. Therefore, communities may underreport it to avoid the stigma and other negative consequences that come with it. Furthermore, studies conducted in Uganda reveal the ecological difference in suicidal rates, ranging from 15 to 99 per 100,000 in various districts^6^–^9^. The post-conflict regions reported higher rates than non-post-conflict areas, and the rates were higher among men than women. A ratio of 4.4:1 for completed suicide for male: female^8^ and 1.7;1 for non-fatal suicidal behavior was reported^7^. Besides gender differences in suicidal rates, researchers document age differences where suicide rates increase with age in Africa^10^. The young people between 15–29 years are at higher risk of suicide than other age groups^11^.

Suicide is a complex behavior consisting of an interplay between many risk factors individual, family, community, and societal levels. The risk factors of suicide among young people may be behavioral disorders, individual characteristics, family, and community factors. The behavioral factors include having to include depressive disorders, substance abuse or dependence, previous suicide attempts, self-injury^12^, and other disorders such as anxiety disorders, eating disorders, conduct disorders^12^–^14^. Examples of individual factors include hopelessness, loneliness^15^, ^16^ social alienation and isolation, lack of belonging, anger, hostility, risky behavior, impulsivity, low stress, and frustration tolerance, poor coping skills, perception of being a burden, adverse/stressful life circumstances, interpersonal difficulties or losses (e.g., relationship breakup, dating violence), school or work problems^12^–^14^ financial issues, previous/current abuse, chronic physical illness or disability and sleep disorders^14^, ^17^. Family factors include a family history of suicide or suicidal behavior, parental mental health problems, previous or current family violence or abuse, family instability or loss, lack of parental support school^12^, ^13^, ^16^.

Lastly, community factors may involve limited access to adequate care for health, mental health, or substance abuse disorders, the stigma associated with seeking care, hostile social and emotional environment, exposure to stigma and discrimination against students, access to lethal means, and exposure to media normalizing suicide^12^, ^16^. It is worth noting that having more than one behavioral or other factors increases the risk of suicide. However, most of our understanding of suicidal behavior among the youth is mainly driven by literature from income countries and little literature from Africa, which may differ due to cultural contexts. Besides, alcohol and other substances are identified as risk factors of suicide and yet the youth have increasedly used them over the years. Therefore it is imperative to explore which factors are associated with suicidality in this already vulnerable population to guide stakeholders on who to prioritize as they intervene to cab this public health problem. The main objective of this study was to describe the prevalence and factors associated with suicidal tendencies among undergraduate university students using alcohol and other psychoactive substances.

## Material and methods

### Setting

The study was conducted among undergraduate students at Makerere University between January to July 2018. Makerere University is the most prominent and one of the oldest institutions of higher learning in Uganda, first established as a technical school in 1922. It consists of nine colleges and one school. It has 12 halls of residence for students with ten at the main campus that occupies an entire hill in Kampala, and two are off-campus. Other students reside in private hostels surrounding the main campus. Convenient sampling was used to identify halls, hostels, and participants. This study involved students who were taking alcohol and other psychoactive substances. The study was mainly conducted in the evening hours and weekends to access more studens in their residences. Four hundred students participated in this study, and this sample size was obtained using the Kish and Leslie sample size formula18, where a 95% confidence interval level was used. The estimated proportion of participants with suicidal tendencies was 50%.

### Data collection

Data was obtained using interviewer-administered questionnaires, which collected socio-demographic characteristics such as age, gender, religion, college of study, employment, and year of study were collected. Specific modules of the Mini International Neuropsychiatric Interview (MINI) tool were used to assess for suicidal tendencies, alcohol use, psychoactive abuse, and dependence. Four hundred study participants were enrolled from known students' housing, which was both on and off-campus. We first screened for those who were undergraduate students and were using alcohol and other psychoactive substances. They were then given additional information regarding the research and its procedure and expectations. The study participants were told that they were free to withdraw from the study at any point. Written informed consent was then obtained from the students who were willing to participate in the study. The researchers addressed any questions that arose. Ethical approval was obtained from the Ethics Committee of the School of Health Sciences, Makerere University. Participants who experienced emotional disturbance were referred to the university hospital for psychological support. Privacy and confidentiality were maintained throughout the study process.

### Measures

The primary outcome of the study was the suicidal tendency, and independent variables of interest were the use of alcohol and other substances and socio-demographics. The M.I.N.I.version 5.0 was used to assess suicidality, use of alcohol, and other psychoactive substances. The MINI is a short structured diagnostic interview developed jointly by psychiatrists and clinicians in the United States and Europe for DSM-IV and ICD-10 psychiatric disorder. This tool has been used in several Ugandan studies to prove cross-cultural adaptation and validation of the self-reporting questionnaire among HIV+ individuals in a rural ART program in southern Uganda^19^. It is divided into modules identified by letters, each corresponding to a diagnostic category. In this study, we used modules C, J, and K that correspond to suicidality, alcohol use, and other substance use.

There were seven specific questions for suicidal tendencies in one month and one item for a lifetime. Answering yes to any of the eight questions was scored for suicidality. Examples of questions included ‘in the past one month did you (i) thik that you would be better off dead or wish you were dead? (ii) think about suicide?

The alcohol module assesses dependence and abuse in the past 12 months. First is a screening question which states, ‘In the past 12 months, have you had three or more alcoholic drinks within a NO YES 3 hour period on three or more occasions?’ If yes, then the participant would continue to answer the subsequent seven questions assessing dependence. Responding yes to 3 or more of those questions qualifies an individual as dependent on alcohol. Four more items were included to evaluate abuse. Answering yes to 1 or more of those questions plus qualification for alcohol dependence qualifies an individual to abuse alcohol.

Module K was extracted to assess the use of other psychoactive substances other than alcohol. This module identifies the psychoactive substance that an individual may be using, followed by seven questions that assess the dependence. Answering yes to 3 or more questions qualifies an individual as dependent on a specific substance they are taking. There were four additional questions to assess substance abuse. One or more yeses on these questions plus qualification for drug dependence qualifies an individual to abuse the substance they are using.

### Data analysis

The data entry stage started immediately after data collection. Data was entered in MS Windows Excel in the form of codes and transferred to the SAS 9.4. Data analysis involved descriptive as well as inferential statistics. At the univariate level, simple descriptive analysis was done for the variables of interest as prevalence rates were calculated. We examined age, gender (male/female), year of study (1^st^–5^th^ year), employment status (employed/unemployed), the region of origin (eastern, northern, western, southern), and religion (Christian/Muslim).

At the bivariate analysis level, the chi-square test was used to assess the strength of association between the independent variables and the dependent variable (suicidal tendency). The variables which were found statistically significant at bivariate analysis with a P-value of less or equal to 0.05 and those that were plausible from documented literature were taken into multivariate logistic regression for further analysis, after which the associated factors were obtained.

## Results

Our study sample comprised of 400 study participants with a mean age distribution of 24 ± 2.26 years. We had a sample distribution of 19.50% and 80.50% of the female to male ratio, respectively. The most predominant religion practiced by the participant is Christianity, with a frequency distribution of 95%. Marijuana or cannabis was noted to be the most predominantly abused psychoactive substance (84.97%). Table 1 presents other univariate demographic variable distributions, and table 2 shows the bivariate demographic distribution relationship by alcohol and substance use.

**Table 1:** Socio-demographic characteristics of study participants

Characteristics	N (%)	P-Value
**Age** 24.0 (SD± 2.26)	400 (100)	
**Gender**		<0.0001
Female	78 (19.50)	
Male	322 (80.50)	
**Tribe**		<0.0001
Central	107 (26.75)	
Western	104 (26.00)	
Eastern	125 (31.25)	
Northern	55 (13.75)	
International	9 (2.25)	
**Religion**		<0.0001
Christian	380 (95.00)	
Muslim	20 (5.00)	
**Drug Type (n=247)**		<0.0001
Stimulants	1 (0.65)	
Cocaine	6 (3.92)	
Narcotics	3 (1.96)	
Hallucinogens	2 (1.31)	
Marijuana	130 (84.97)	
Tranquilizers	1 (0.65)	
Miscellaneous	10 (6.54)	
**Employment Status**		<0.0001
Employed	106 (26.50)	
Unemployed	294 (73.50)	
**Year of study**		<0.0001
1st	32 (8.00)	
2nd	127 (31.75)	
3rd	153 (38.25)	
4th	81 (20.25)	
5th	7 (1.75)	
**Faculty**		<0.0001
Tech & Agric	65 (16.25)	
Education, Humanities & Law	172 (43.00)	
Sciences & Vet Medicine	163 (40.75)	
**Alcohol Dependence**		<0.0001
Dependent	248 (62.00)	
Not dependent	152 (38.00)	
**Alcohol Abuse**		<0.0001
Yes	301 (75.25)	
No	99 (24.75)	
**Substance Dependence**		<0.0001
Yes	60 (15.00)	
No	340 (85.00)	
**Substance Abuse**		<0.0001
Yes	106 (26.50)	
No	294 (73.50)	
**Suicide attempt**		<0.0001
yes	08 (2.00)	
No	392 (98.00)	
**Deliberate Injury**		<0.0001
Yes	12 (3.00)	
No	388 (97.00)	
**Took active step for suicide**		<0.0001
Yes	6 (1.50)	
No	394 (98.50)	
**Had Suicide Plan**	1 (0.25)	<0.0001
Yes	399 (99.75)	
No		

**Table 2:** Descriptive of Substance abuse and Alcohol abuse by socio-demographic

Characteristics	Alcohol abuse N=301(%)	P-Value	Substance Abuse N=106 (%)	P-Value
**Gender**				
Female	61(20.27)	0.50	24(22.64)	0.34
Male	240(79.73)		82(77.36)	
**Tribe**				
Central	69(28.90)	0.01	27(25.47)	0.19
Western	87(23.90)		32(30.19)	
Eastern	93(30.90)		29(27.36)	
Northern	46(15.28)		18(16.98)	
International	06(1.99)		00	
**Religion**				
Christian	296(98.34)	<0.001	97(91.51)	0.06
Muslim	05(1.66)		09(8.49)	
**Employment status**				
Employed	213(70.76)	0.03	73(68.87)	0.21
Unemployed	88(29.24)		33(31.13)	
**Year of study**				
1st	22(7.31)	0.01	05(4.72)	0.09
2nd	106(35.22)		35(33.02)	
3rd	117(38.87)		50(47.17)	
4th	50(16.61)		15(14.15)	
5th	06(1.99)		01(0.94)	
**Faculty**				
Technology & Agriculture	49(16.28)	0.99	18(16.98)	0.84
Education, Humanities & Law	129(42.86)		43(40.57)	
Sciences & veterinary Medicine	123(40.86)		45(42.45)	

The Prevalence of suicidality was 6.3%, where 25 out of 400 participants had suicidal tendencies. Its distribution by gender was 5.13% and 5.30% for females and males respectively. Bivariate analysis of the region of origin and suicidality showed a prevalence rate of 5.61% for central, 6.73% for western, 2.40% for eastern, and 16.36% for northern. Among those who had substance dependence, suicidality was at 13.33%, while participants busing other substances had a prevalence of 10.38%. For suicidal tendencies among alcohol use, those with alcohol dependence had a prevalence rate of 7.29% while the alcohol abuse prevalence rate was 6.33%. Table 3 shows other demographic suicidal tendencies prevalence rates.

**Table 3:** Descriptive characteristics of Students by Suicidality prevalence

Characteristics	Suicidality n=25(%)	P-Value
**Gender**		
Female	06(7.69)	0.56
Male	19(5.92)	
**Region of origin**		
Central Uganda	06(5.61)	0.01
Western Uganda	07(6.73)	
Eastern Uganda	03(2.40)	
Northern Uganda	09(16.36)	
**Religion**		
Christian	25(6.60)	0.24
Muslim	00(00)	
**Employment status**		
Employed	02(1.90)	0.03
Unemployed	23(7.82)	
**Year of study**		
1st	01(3.13)	0.25
2nd	04(2.15)	
3rd	11(7.24)	
4th	08(9.88)	
5th	01(14.29)	
**Faculty**		
Technology & Agriculture	5 (7.69)	0.87
Education, Humanities & Law	10 (5.85)	
Sciences & Veterinary Medicine	10 (6.13)	
**Alcohol Dependence**		
No	7 (4.61)	0.28
Yes	18 (7.29)	
**Alcohol abuse**		
No	6 (6.06)	0.92
Yes	19 (6.33)	
**Substance Dependence**		
No	17 (5.01)	0.01
Yes	8 (13.33)	
**Substance abuse**		
No	14 (4.78)	0.04
Yes	11 (10.38)	

The p-value for odds ratio estimates at bivariate analysis showed a statistically significant association between employment status and suicidal tendency. Being employed was protective of suicidality (OR=0.229, 95% CI= 0.053–0.998, P=0.05). Similarly, being a student in the third year and above was associated with suicidal tendencies (OR=1.667, 95% CI= 1.059- 2.624, P=0.03). Psychoactive substance use exhibited a statistically significant association with suicidal tendency as being dependent on marijuana or cannabis (OR= 2.914, 95% CI= 1.197- 7.095 P=0.02) was associated with suicidal tendency; and psychoactive substance abuse (OR= 2.308, 95% CI= 1.013–5.257, P=0.04) was associated with suicidal tendency as well. Being dependent on both alcohol and other psychoactive substance had a statistically significant association with suicidality (OR= 3.439, 95% CI= 1.350–8.761 P=0.01). Other binary logistic regression analysis showed that suicidal tendencies among our participants did not have an association with age, gender, religious inclination, and any of the alcohol use statuses as they were statistically non-significant. See Table 4 for other odds ratio estimates of suicidal tendency association with covariates.

**Table 4:** Bivariate and multivariate analysis showing association between independent variables and suicidal tendency

Characteristics	^∞^COR (95%Cl)	P-Value	^*^AOR (95%Cl)	P-Valve
**Age** (SD)	0.985(0.823–1.180)	0.87		
**Gender**				
Female	Ref			
Male	0.755(0.291–1.958)	0.56		
**Year of study**				
≤ 2years	Ref			
>2 years	1.667(1.059–2.624)	0.03		
**Drug Types**				
Others	Ref			
Marijuana	1.429(0.624–3.274)	0.40		
**Employment Status**				
Unemployed	Ref			
Employed	0.229(0.053–0.998)	0.05		
**Alcohol Dependence**				
No	Ref		Ref	
Yes	1.628(0.664–3.995)	0.29	2.043 (0.812–5.137)	0.1290
**Alcohol Abuse**				
No	Ref		Ref	
Yes	1.048(0.406–2.703)	0.92	1.223 (0.466–3.212)	0.6822
**Substance Dependence**			Ref	
No	Ref			
Yes	2.914(1.197–7.095)	0.02	2.900 (1.154–7.286)	0.0235
**Substance Abuse**				
No	Ref		Ref	
Yes	2.308(1.013–5.257)	0.04	2.711 (1.155–6.364)	0.0220
**Alcohol &** **other Substance dependence**				
No	Ref		Ref	
Yes	3.439(1.350–8.761)	0.01	3.573 (1.356–9.419)	0.0100
**Alcohol & other** **substance abuse**				
No	Ref		Ref	
Yes	1.757(0.732–4.221)	0.21	2.200 (0.888–5.453)	0.0886

^*^AOR- Adjusted Odd Ratio

^∞^COR-Crude Odd ratio

For the multivariate logistic regression, we adjusted for age, gender, year of study of the participant, and employment status across the different alcohol and other substance use status. There was no statistically significant association for both alcohol use status and suicidal tendencies. Psychoactive substance use other than alcohol had a statistically significant association with suicidal tendency as being dependent on marijuana or cannabis (P=0.02, AOR= 2.900, 95% CI= 1.154–7.286) was associated with suicidal tendency; similarly, psychoactive substance abuse (P=0.02, AOR= 2.711, 95% CI= 1.155- 6.364) was also associated with suicidal tendency as well. Being dependent on alcohol and other substances had a statistically significant association with a suicidal tendency (P=0.01, AOR= 3.573, 95% CI= 1.356- 9.419). For participants who abused both alcohol and other substances, there was no statistically significant association after controlling for the confounding factors.

## Discussion

This study aimed to establish the proportion of suicidal tendencies and factors associated with it among students using alcohol and other psychoactive substances. The key findings in this study were 6.3% of the study population had suicidal tendencies, which showed an increase by the year of study. Our study also showed that suicidality was statistically significantly associated with substance abuse, dependence, and dependence on alcohol and other psychoactive substances. It is noteworthy to mention that students with any form of employment had lower odds of suicidal tendencies than unemployed students. And the odds estimate for marijuana as the substance of use showed a probable trend in association with increased suicidal tendencies.

The proportion of the study participants with suicidality is lowered compared to the national Prevalence of 9.9%^20^. Similarly, the rate of suicidality was lower than the 9.5% reported among South African youth^21^. Our study participants could have underreported their suicide behavior as it is highly stigmatized in the communities and considered a criminal act^22^, ^23^. Legally, for those who have failed suicide attempts, they may be prosecuted and jailed for their action^24^. Secondly, our study participants could have been more guarded because they were also taking other psychoactive substances considered illegal in the country. These factors could have made it harder for them to talk about their suicidal behaviors during their interview in this study freely. On the other hand, there is a lot of community violence in South Africa that contributes to childhood adversities, which are a risk factor to suicidal behavior among the youth^25^. Therefore, more specialized strategies should be applied in future studies while discussing sensitive topics such as suicidal behavior and substance abuse to get accurate estimates among these vulnerable groups.

Suicidal behavior was statistically significantly associated with substance abuse and dependence. This finding supports evidence in the literature about the increased risk of suicide mortality26. It is important to note that most study participants (85%) were using marijuana or cannabis. Females were more likely to abuse cannabis and alcohol, supporting evidence from other studies^26^,^27^. This is not surprising because marijuana or cannabis is cheap and readily available in a Ugandan setting compared to other psychoactive substances^28^. However, the most exciting finding of our study is the fact that marijuana or cannabis use had an association with suicidal tendency though the temporality cannot be ascertained. This is important, especially with the new trend of withdrawing the ban on marijuana or cannabis use in most developed nations of the world viz-a-viz the futuristic global prevalence of suicide cases in few years to come^29^. Other researchers have documented the strong relationship between suicidal behavior and substance abuse and dependence among the youth; for example, Pompili et al. (2012) reviewed the literature on adolescents and documented a strong relationship between suicide and any substance abuse disorder^30^. Some researchers have questioned whether SUD plays a proximal or distal risk to suicide^31^. Our study findings add evidence to this area from an African perspective. Therefore, health workers should screen for suicidality when interacting with youth who take alcohol and other psychoactive substances. Psychosocial support should be readily accessible to students to help with any mental health issues.

Suicidal behavior was associated with unemployment and increased years at the university. Notably, both being unemployed and finishing college is associated with increased stress^32^; for example, the students in the final years are more likely to be stressed about finishing their programs and uncertain of the future. Alternatively, those in other years become more concerned about their academic performance as time goes. Moreover, the more years the students spend at the university, the more chance they would explore relationships, which may sometimes come with stressful events^33^,^34^. Besides, being unemployed brings about financial dependence, which is sometimes stressful. Therefore, increased stress may facilitate the youth to cope by abusing substances^35^. It is also interesting to note that students who have more suicide behavior were from the northern part of the country, which had a recent history of civil war that lasted more than 20 years. This finding could explain both proximal and distal risk of other underlying psychological distress resulting from the exposure to trauma^36^, ^37^. Simultaneously, being in the post-conflict area comes about with a lot of stress due to several losses, and some people cope by abusing substances or alcohol^36^. Therefore, both stress and psychopathology may increase the risk of suicide in the communities^38^.

## Study limitations

First, the sampling method and questionnaire structure are cross-sectional, with many self-report variables subject to bias. Convenient sampling was used in this study, and therefore, the findings may not be generalizable. Additionally, our study may be prone to social desirability bias due to the probability of under-reporting suicidality due to endemic socio-cultural norms in Uganda. However, this study highlights essential findings that encourage the public to implement preventive strategies to help the youth who may be abusing the substances before they become suicidal.

It is also noteworthy that the findings from this study have public health significance. Understanding the role of alcohol and psychoactive influence towards suicidal possibilities among the youth helps comprehend the need for further research. These health and government policy advocacy adjustments are based on the vulnerable population. The findings of this study may be used to develop strategies geared towards reducing alcohol and other psychoactive substance abuse among the youth in the university settings, Uganda, and the developing nations in Africa at large. The results of our study may also be beneficial to strengthen strategies or additional incentives to develop effective substance abuse quit programs, which may translate to a reduction of the development of mental illness and suicidality among the youth. Educational institutions need to strengthen or establish psychosocial support centers and encourage utilization among students. The findings of this study will also add to the body of knowledge regarding suicidality among the youth who abuse alcohol and substances in low and middle-income countries. We, therefore, recommend that strategies that target prevention of substance dependence among the youth be implemented and strengthened in higher institutions of learning.
